# Enhancement of Medical Interns' Levels of Clinical Skills Competence and Self-Confidence Levels via Video iPods: Pilot Randomized Controlled Trial

**DOI:** 10.2196/jmir.1596

**Published:** 2011-03-01

**Authors:** Margaret Hansen, George Oosthuizen, John Windsor, Iain Doherty, Samuel Greig, Karina McHardy, Lloyd McCann

**Affiliations:** ^7^Department of Performance ImprovementOxford Radcliffe Hospitals NHS TrustOxfordUnited Kingdom; ^6^Department of Public HealthUniversity of OxfordOxfordUnited Kingdom; ^5^Private practiceChristchurchNew Zealand; ^4^Learning Technology UnitFaculty of Medical and Health SciencesThe University of AucklandAucklandNew Zealand; ^3^School of MedicineDepartment of SurgeryThe University of AucklandAucklandNew Zealand; ^2^Edendale HospitalDepartment of SurgeryPietermaritzburgSouth Africa; ^1^School of NursingAdult Health DepartmentUniversity of San FranciscoSan Francisco, CAUnited States

**Keywords:** Medical education, healthcare, mobile technology, urinary catheterisation

## Abstract

**Background:**

Designing and delivering evidence-based medical practice for students requires careful consideration from medical science educators. Social Web (Web 2.0) applications are a part of today’s educational technology milieu; however, empirical research is lacking to support the impact of interactive Web 2.0 mobile applications on medical educational outcomes.

**Objectives:**

The aim of our study was to determine whether instructional videos provided by iPod regarding female and male urinary catheter insertion would increase students’ confidence levels and enhance skill competencies.

**Methods:**

We conducted a prospective study with medical trainee intern (TI) participants: 10 control participants (no technological intervention) and 11 intervention participants (video iPods). Before taking part in a skills course, they completed a questionnaire regarding previous exposure to male and female urinary catheterization and their level of confidence in performing the skills. Directly following the questionnaire, medical faculty provided a 40-minute skills demonstration in the Advanced Clinical Skills Centre (ACSC) laboratory at the University of Auckland, New Zealand. All participants practiced the skills following the demonstrations and were immediately evaluated by the same faculty using an assessment rubric. Following the clinical skill evaluation, participants completed a postcourse questionnaire regarding skill confidence levels. At the end of the skills course, the intervention group were provided video iPods and viewed a male and a female urinary catheterization video during the next 3 consecutive months. The control group did not receive educational technology interventions during the 3-month period. At the end of 3 months, participants completed a follow-up questionnaire and a clinical assessment of urinary catheterization skills at the ACSC lab.

**Results:**

The results indicate a decline in skill competency over time among the control group for both male and female catheterizations, whereas the competency level was stable among the experimental group for both procedures. Interaction results for competency scores indicate a significant level by group and time (*P* = .03) and procedure and group (*P* = .02). The experimental group’s confidence level for performing the female catheterization procedure differed significantly over time (*P* < .001). Furthermore, confidence scores in performing female catheterizations increased for both groups over time. However, the confidence levels for both groups in performing the male catheterization decreased over time.

**Conclusions:**

Video iPods offer a novel pedagogical approach to enhance medical students’ medical skill competencies and self-confidence levels. The outcomes illustrate a need for further investigation in order to generalize to the medical school population.

## Introduction

Medical students obtain a rich learning experience in the Advanced Clinical Skills Centre (ACSC) at The University of Auckland, New Zealand. They take courses such as crisis management in anesthesia, open and laparoscopic surgical skills, advanced cardiac life support, general surgical skills, gynecologic principles, orthopedics, and general physician assessment skills. Faculty members at the ACSC provide students with modern human-patient simulations as a part of the medical education curriculum and are willing to try new medical pedagogy to enhance learning outcomes.

The traditional apprenticeship model for teaching clinical skills is no longer feasible due to the shortage of faculty [1]; however, this educational model may be augmented with simulation training, centralized skills training centers, and Web/Medicine 2.0 applications [2,3] that support a constructivist approach to learning [4]. Enhanced medical trainee education, by using new and emerging technologies for teaching and learning, may increase team communication and collaboration, skill engagement and performance, and competency with consequent adverse events, or preventing medical errors. Furthermore, there is an ethical imperative to ensure optimal treatment without doing harm to patients and, therefore, technological advancements may provide a safe environment for medical students to practice [5]. The aim of this experimental study was to determine whether female and male urinary catheter insertion videos provided via video iPods would increase medical students’ confidence levels and enhance skill competencies.

### Background

Medical video technology delivered via a mobile device has great potential for cultivating a positive learning landscape in medical schools. Educators know that millennial students, born between 1980 and 1994, are technologically adept, stressed, high-achieving, confident, and self-assured [6]. These students demand convenience and require specific educational direction and guidance while attending college. Therefore, the introduction of mobile audiovisual technologies for this type of student is ideal because of the convenience they provide and the specific educational content they deliver. These students are accustomed to waking up and automatically having the current medical news, viewpoints, research, and education to listen to and perhaps view while commuting to the university, work, or the gym. This is made possible by podcasts and video mobile technologies.

### Conceptual Framework

From an educational theoretical perspective, Mayer’s cognitive theory of multimedia learning posits that people learn best when images are combined with words in an electronic learning environment [7]. Mayer’s definition of multimedia includes animation and narration, not just corresponding text and static illustrations. Mayer’s studies involve the use of short multimedia tutorials and result in significant learning outcomes. Therefore, Mayer’s theory of multimedia learning served as the conceptual framework for this study.

### Purpose

The aim of this investigation was to determine whether female and male urinary catheter insertion videos provided via video iPods would increase medical students’ confidence levels and enhance skill competencies and retention. It is important to measure both confidence and competence because self-confidence is not always a reliable indicator of skill competence. This study was designed around a central hypothesis that medical students who receive medical skill videos delivered via a mobile technology would have increased self-confidence and skill competency.

### Literature Review

Students in the health care professions have benefited from repeatedly listening to learning material at their convenience via mobile technology and have reported high satisfaction using audio and video formats in learning [8,9]. Social Web (Web 2.0) applications, such as podcasts/vodcasts, are becoming common technology applications in health care professional education, and novel research is being conducted and published regarding learning outcomes [10-12]. While no one has clearly defined and agreed on what Medicine or Health 2.0 is, researchers have determined the term originated from the concepts of medicine and Web 2.0 [13]. A podcast/vodcast may consist of an audio and/or video file distributed to a selected media player over the Internet, some smart phones, and iPad-style notebooks, or downloaded to an iPod-like device. Video podcasts may then be referred to as Medicine/Health 2.0 tools to affect health care and education, and perhaps even in underserved countries where mobile health technology is expanding [14].

In regard to use of video iPods in higher education, audio and video formats prove effective in enhancing learning outcomes [14-17]. Health care professional students report satisfaction in listening to lecture material and viewing clinical skills [16]. This innovative pocket-sized mobile device is becoming part of physicians’ repertoire of diagnostics, educational tools, and research interventions. However, there is a paucity of evidence around how mobile technology, and using it as a platform for medical education, affects practitioner competence in clinical skills and procedures.

### Research Questions

Urinary catheterization of male and female patients is an important technical skill requiring practice. Therefore, we formulated research questions and invited a cohort of medical trainee interns (TIs) attending a clinical skills rotation at ACSC to participate in the study. We set out to answer the following questions. What are TIs’ levels of confidence in performing male and female urinary catheterizations prior to a clinical skills course? What are TIs’ levels of competence and confidence in performing female and male urinary catheterization after a clinical skills course? What are TIs’ levels of competence and confidence in performing male and female urinary catheterization (comparing the control and intervention groups) at 3 months after a clinical skills course? These questions are critical given that urinary catheterization may be a contributing factor to nosocomial infections and other medical conditions [18,19].

## Methods

### Sample

This was a prospective study using a random sample. Carrying out a prospective study with randomized participants in medical education is challenging for three reasons: (1) a randomized trial, particularly if a control group is involved, is perceived to put some students at a disadvantage, (2) variables are difficult to control, and (3) appropriate outcome measures are difficult to identify [20]. We designed and carried out the study with these reasons in mind in order to control for confounding variables.

**Figure 1:**
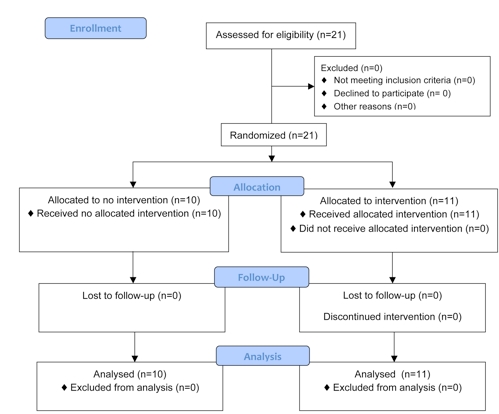
CONSORT flow diagram

The study sample consisted of 21 final-year medical TIs attending the University of Auckland’s School of Medicine (see Figure 1). Medical TI participants who completed 6 years of medical training were randomized into two groups. The control group (n = 10) and the intervention group (n = 11) were enrolled in a clinical skills course lasting 2 consecutive days. All TIs are required to complete the clinical skills course during the final year of medical school. Six clinical-skill course cycles are held throughout the year, and we randomly selected this study sample from the six cycles of TIs rotating through the scheduled courses in 2006.

Since the participants were enrolled in an established teaching course, Institutional Review Board of Protection of Human Subjects approval was not required. Furthermore, trials including health care providers as participants are exempt, and therefore this randomized control trial was not registered [21]. We obtained consent to participate in the study from the medical TI participants.

A power analysis was not conducted for this experimental study. TIs were assured that participation in the study was voluntary and would not have an effect on any course grades or employment at the University of Auckland.

### Precourse Evaluation: Skill Experience

The skills taught during the clinical skills course were advanced cardiac life support, lumbar puncture procedure, nasogastric tube insertion, urinary catheterization of a male and a female patient, and the care of an open contaminated wound. Participants were asked at the onset to state expected learning outcomes from the clinical skills course and at the end of the study to evaluate the course. Precourse questionnaires were newly developed and distributed by the medical school faculty to both groups of TIs before instruction on male and female urinary catheterization. The precourse evaluation questionnaire contained five questions concerning (1) prior exposure to the male and female urinary catheterization skill, (2) self-reported level of confidence in performing the skills, (3) importance of mastering the procedures for male and female urinary catheterizations, (4) to what extent TIs would make use of the clinical procedure in future medical practice, and (5) TIs’ level of fatigue before receiving training for the medical skills.

Another purpose of the precourse questionnaire was to determine levels of TIs’ preexisting knowledge that might confound the results of the study [20]. Validity and reliability of the newly developed questionnaire were not assessed. All questionnaires were collected from the TIs by the clinical skills course director and kept in the director’s locked office cabinet. Participants were assured that their precourse questionnaire responses would be kept confidential and that the data would be kept for 5 years before being securely disposed of.

### Competency and Confidence Levels

Directly after completing the precourse questionnaire, the TIs viewed a professionally filmed 15-minute male urinary catheter insertion video and a 15-minute female urinary catheter insertion video in the ACSC on a television monitor. Immediately following the videos, the TIs viewed a live 10-minute demonstration of the male and female urinary catheterization skill (5 minutes for each skill) performed on a mannequin by a physician in the ACSC. The total training time was approximately 40 minutes. The TIs were directly given the opportunity to practice each skill on the male and female mannequins in the ACSC under the supervision of clinical tutors. All TIs participated in the practice session. Each practice procedure lasted on average 10 minutes.

The TIs then completed both procedures while being formally assessed by a physician faculty member who used a 16- and 15-item paper-based competency skill evaluation tool for the male and female catheterization procedures, respectively. An ACSC faculty physician wrote the competency skill evaluation tools. The assessment tool consisted of a written skill checklist, comprising direct observation with criteria, for steps in the demonstrated/learned procedures. Kneebone [22] recognizes that there “is as yet no uniform approach to measuring performance”; however, we chose this method because we did not consider the clinical skills to be sufficiently complex to warrant a more arduous form of assessment. The reliability and validity for this form of assessment are high for surgical skills procedures, since subjectivity is removed from the evaluation process [23]. Each participant had to satisfactorily demonstrate insertion of a urinary catheter into the male and female simulation mannequins while being assessed against a set of standardized procedure metrics.

The students’ competency scores were calculated by averaging the 16 clinical procedural steps for the male catheterization skill and the 15 clinical procedural steps for the female catheterization. The procedural steps were setting up a sterile field; maintaining sterility during urinary catheter tray setup; choosing the correct size and type of catheter; explaining the procedure to the patient; questioning the patient about allergies to iodine, latex, and adhesives; washing hands before donning sterile gloves; demonstrating correct technique for donning sterile gloves; cleansing the patient’s skin while maintaining aseptic technique; properly draping the patient with a sterile towel; inserting the catheter while adhering to sterile technique; ascertaining correct depth of catheter insertion before inflating the Foley catheter balloon; gently pulling back on the catheter to confirm balloon inflation and position; correctly connecting and anchoring the catheter; indicating that patient’s foreskin would be replaced at the end of the procedure; and properly documenting the patient’s chart (date of insertion, volume of balloon inflation, and any complications). The student either “correctly” or “incorrectly” performed each step of the procedure.

**Figure 2:**
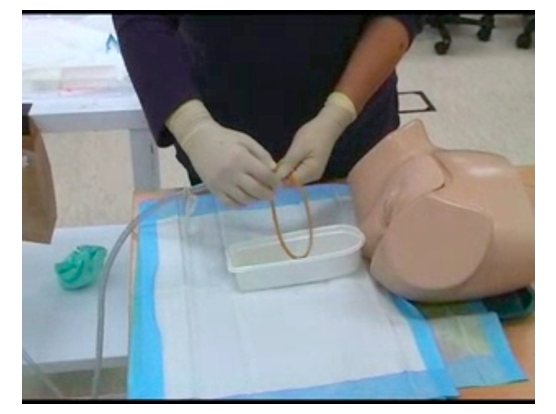
Advanced Clinical Skills Centre faculty-prepared video of a female catheterization (University of Auckland, New Zealand)

Following this evaluation process, TIs were randomly assigned to two groups: control and intervention. The randomization process involved consecutively numbering 21 envelopes, provided by a third party not involved in conducting the study, with allocation to either the intervention (video iPod) or control group (no technology) in each envelope. After consenting to participate in the study, TIs were irreversibly randomized by being provided the sealed envelope. The intervention group was given unlimited access to two ACSC faculty-prepared videos (see Figure 2) presented via a video iPod: female (Multimedia Appendix 1) and male urinary catheterization procedures (Multimedia Appendix 2). Use of the video iPod was explained to the intervention group, and members of the intervention group were contacted by email 2 weeks after receiving the video iPods to inquire about technical difficulties using the hardware or software. To avoid a confounding variable, the control group was not given any technological intervention for 3 months. The intervention group was asked not to share the videos delivered via the video iPods with their peers or discuss the learning process.

After 3 consecutive months, all participants were reevaluated against the same set of standardized metrics used before the intervention and by the same evaluators who conducted the training course to ascertain students’ competency in the urinary catheterization skills. Evaluators were blinded to which group was being assessed. TIs filled out a 3-month follow-up course questionnaire that measured exposure to the male and female urinary catheter procedures since attending the initial clinical skills course at the ACSC and their confidence levels (secondary end point) for each catheterization procedure. The ACSC staff member researcher responsible for arranging the follow-up meeting used text messaging to remind participants of the upcoming 3-month follow-up evaluation at the ACSC.

## Results

### Sample

Data were entered into SPSS version 18 (IBM Corporation, Somers, NY, USA) for statistical analyses. Demographic data (see Table 1) were entered and a male/female catheterization experience variable was calculated.

**Table 1:** Demographic characteristics of trainee interns (N = 21)

Characteristic		Intervention group (n = 11)	Control group (n = 10)
Males		5	2
Females		6	8
**Age range (years)**			
	16-26	8	9
	27-36	3	0
	37-46	0	1

### Precourse Evaluations: Skill Experience

Before learning the clinical skill procedure, students’ experience was measured as the number of times they had performed a urinary catheterization. For the 3-month follow-up experience measurement, the number of times students had “performed” or “assisted” with the clinical procedure was included in the data collection. Table 2 shows results for skill experience with male and female catheterization procedures. For the control group, we did not obtain data for one participant at the 3-month follow-up on both procedures. For the intervention group, we obtained data for 10 participants at the precourse and 3-month follow-up times. For one question at the 3-month follow-up time, we obtained only eight responses.

**Table 2:** Trainee interns’ urinary catheterization skill experience in terms of the number of times they had seen, assisted in, or performed the procedure

Time point of study		Catheterization procedure	Control group					Intervention group				
			0	1-2	3-4	>4	Total n	0	1-2	3-4	>4	Total n
**Before course**												
	Preintervention	Male	0	7	3	0	10	2	5	2	1	10
	Preintervention	Female	0	8	2	0	10	2	6	0	2	10
**Seen since course**												
	3-month follow-up	Male	1	4	4	0	9	2	5	2	1	10
	3-month follow-up	Female	3	3	1	2	9	5	4	0	1	10
**Assisted since course**												
	3-month follow-up	Male	3	6	0	0	9	5	2	1	0	8
	3-month follow-up	Female	6	3	0	0	9	9	0	0	1	10
**Performed since course**												
	3-month follow-up	Male	1	4	1	3	9	7	0	1	2	10
	3-month follow-up	Female	6	2	1	0	9	7	2	0	1	10

### Competency and Confidence Levels

A high procedural score suggests higher TI competency. The results indicate a decline in competency over time in the control group for both female and male catheterizations. However, the competency level was stable in the intervention (video iPod) group for both procedures. The postcourse male urinary catheter insertion competency scores were nearly equal for the control and intervention groups and close in value to the postcourse female urinary catheterization competency scores for the control group. The greatest difference was in the intervention group competency score for the female catheterization procedure at the 3-month follow-up course, which was higher than the other competency scores (see Table 3).

The TIs’ confidence levels were measured at the pre-, post-, and 3-month follow-up course for female and male catheterizations separately. The mean values for the participants are reported in Table 3. The change scores indicate a practical significance and perhaps, with a larger sample size, the results would have been different and deemed clinically significant.

**Table 3:** Trainee interns’ skill competency and confidence scores by procedure (N = 21)

		Time point of study	Control group mean (SE)^a^	Intervention group mean (SE)
**Skill competency by procedure**				
	Female catheterization	Postcourse	1.79 (0.07)	1.88 (0.07)
		3-month follow-up course	1.61 (0.07)	1.88 (0.08)
		Change score	0.18	0.00
	Male catheterization	Postcourse	1.83 (0.07)	1.79 (0.07)
		3-month follow-up course	1.69 (0.70)	1.80 (0.07)
		Change score	0.14	0.01
**Skill confidence by procedure**				
	Female catheterization	Precourse	2.51 (0.32)	3.36 (0.35)
		Postcourse	2.64 (0.32)	3.59 (0.35)
		3-month follow-up course	3.61 (0.32)	4.50 (0.35)
		Change score	1.10	1.14
	Male catheterization	Precourse	3.94 (0.32)	4.60 (0.35)
		Postcourse	3.84 (0.34)	4.31 (0.37)
		3-month follow-up course	2.89 (0.34)	3.93 (0.35)
		Change score	1.05	0.67

^a^ SE: standard error.

As noted in the group-by-time interaction (control vs intervention by post- vs 3-month follow-up course), the intervention competency scores were stable for male and female catheterization procedures, while the control group competency scores for female and male catheterizations became smaller. Possible interactions between variables were investigated by using the Kenward-Roger autoregressive correlation matrix. The calculated interaction effect for the interaction between group (control vs intervention), procedure (female vs male catheterization), and time (pre-, post-, 3-month follow-up course) was not significant at the .05 level. However, the group-by-time interaction and the procedure-by-group interaction were significant at the .05 level. The results are highlighted in Table 4.

**Table 4:** Interaction results for trainee interns’ competency scores (N = 21)

Interaction variables	Analysis of variance	*P* value
Group by procedure by time	F_1,27.4_ = 0.05	.83
Group by time	F_1,27.6_ = 5.55	.03
Procedure by group	F_1,21.1_ = 7.39	.02

In regard to TI confidence levels, there was no statistically significant interaction between group (control vs intervention), procedure (female vs male catheterization), and time (post- vs 3-month follow-up course). Neither was there a statistically significant interaction between group (control vs intervention) and time (pre- vs 3-month follow-up course). However, there was a statistically significant interaction between procedure (female vs male catheterization) and time (pre- vs 3-month follow-up course). Over time the confidence scores in performing the female catheterization procedure increased for both groups, while the confidence scores in performing the male catheterization procedure decreased. The results are presented in Table 5.

**Table 5:** Interaction effects for trainee interns’ catheterization skill confidence levels (N = 21)

Interaction variables	Analysis of variance	*P* value
Group by procedure by time	F_2,53.7_ = 0.49	.62
Group by time	F_2,55.4_ = 0.33	.72
Procedure by time	F_2,56.5_ = 19.13	<.001

## Discussion

### Principal Findings

Urinary catheterization skill competency declined over time in the control group for both male and female catheterizations, whereas the competency level was stable in the intervention group for both procedures. This finding is consistent with findings from other studies where a particular skill or procedure is not practiced: competency is likely to diminish over time. The study cohort of final-year medical TIs also would not have the opportunity to perform urinary catheterization on a regular basis for various clinical reasons [24,25]. Interaction results for competency scores indicate a significant level by group and time, and by procedure and group. The intervention group’s confidence level for performing the female catheterization procedure differed significantly over time. Furthermore, TIs’ confidence scores in performing female catheterizations increased in both groups over time. However, the confidence scores for both groups in performing the male catheterization decreased over time.

From a clinical education point of view (patient anatomy and comfort level of the TI participant), the male procedure may have been more challenging for the female medical students, who constituted the majority of the study’s sample. If gender is a factor and has potential effects on performance and safety, then more research needs to be conducted. In regard to evaluating the student’s gender with respect to male and female patients in the clinical setting, anecdotal evidence from researchers’ experiences highlights this as a potential educational issue. Also, data suggest a trend for the intervention group to access the two videos via the video iPods just before the 3-month follow-up assessment period and not throughout the 12 weeks following the instruction and immediate postcourse assessment. In the future, researchers may want to measure time-on-viewing (also called time-on-learning) the videos and assess where the students viewed the videos. Time-on-viewing is a significant factor in learning a skill and may be logged in a personal journal by the learner [26]. Learning space is also a factor affecting learning, and it may be beneficial to analyze the effects of time-on-viewing and location-of-learning on medical students’ competency, confidence, and knowledge-retention levels.

The significance of implementing mobile devices as learning tools needs to be further evaluated for future educational purposes. Developers of computer-based instruction and other learning technologies benefit from gathering information from formative evaluation strategies when developing instructional media in order to assess clinical judgment and practice [27]. Hence, it may be helpful to conduct a pilot study with the goal of determining which media format is most beneficial for medical students’ learning and demonstrating clinical skills before making a final decision about adopting these and other popular mobile social learning technologies. Furthermore, it is recommended that the educator provide the listener/viewer with some sort of interactivity option with the podcast/vodcast producer(s) to answer questions or provide user feedback regarding the podcast/vodcast [28].

### Limitations

Even though the statistically significant improvement in student confidence levels for performing the female urinary catheterization clinical skill over time was positive, the primary limitation of this study is the small sample size. Other concerns that need to be taken into consideration in medical education research include: technical requirements and potential problems that students might encounter with video iPods; pressure to perform the skills in a specific way because medical faculty are conducting a study of clinical skill proficiency; and the effect of time-on-viewing and location-of-learning these clinical skills via the video iPod on medical students’ competency and confidence levels.

### Conclusion

The use of mobile devices, such as a video iPod, smart phone, or iPad-like notebook, for health care professional education is interesting and promising. A convenient and ubiquitous mobile technical device providing scenario-based video presentations enhances a constructivist approach to medical education. There are many potential educational uses of the video iPod for patients, families, and their health care providers all over the world. Furthermore, it is beneficial to offer the audience viewing the videos on mobile devices some interaction with the devices and/or to provide feedback from the podcast/vodcast presenter [28]. The results of this small experimental study suggest that video iPods enhance medical students’ confidence in performing the female urinary catheterization procedure over time in a learning resource center, and medical students’ skill competency levels remained the same over time when they accessed the video demonstrations, via a video iPod, of the medical procedure. Social learning technologies, such as the video iPod, engage learners and provide an environment where students may connect, cultivate ideas, and collaborate while learning important clinical skills, in keeping with the concept of Medicine 2.0 [29]. Learning outcomes are influenced by mobile technology and offer a convenient adjunct to traditionally designed medical school courses.

## Multimedia Appendix 1

Female catheterization

## Multimedia Appendix 2

Male catheterization

## Abbreviations

ACSC: Advanced Clinical Skills Centre
TI: trainee intern
